# Involvement of miR-770-5p in trastuzumab response in HER2 positive breast cancer cells

**DOI:** 10.1371/journal.pone.0215894

**Published:** 2019-04-22

**Authors:** Senem Noyan, Hakan Gurdal, Bala Gur Dedeoglu

**Affiliations:** 1 Biotechnology Institute, Ankara University, Ankara, Turkey; 2 Department of Medical Pharmacology, Faculty of Medicine, Ankara University, Ankara,Turkey; University of South Alabama Mitchell Cancer Institute, UNITED STATES

## Abstract

miRNAs may play effective roles in breast cancer so modulating their expression levels could have therapeutic benefits. Recent studies have found the combination of miRNA-based therapeutics with conventional drugs as promising. This study aimed to find drug-responsive miRNAs, and explore their anticancer activities in HER2+ breast cancer cells and regulatory role in the trastuzumab response. qRT-PCR-array analysis was performed with effective concentrations of tamoxifen and trastuzumab treated BT-474, SK-BR-3 and MCF-7 cells. Motility and invasion analyses were performed with wound healing and xCELLigence impedance-based assays respectively. Viability of cells following mimic transfection and drug treatment was assessed by WST-1 assay. Western blot analysis was used to assess miR-770-5p regulation of proteins and their phosphorylated forms. The clinical relevance of miR-770-5p was examined by TCGA data analysis. The qRT-PCR-array results indicated that miR-770-5p was responsive in a drug and cell line independent manner. Overexpression of miR-770-5p inhibited the motility and cell invasion through regulation of AKT and ERK proteins. Additionally, miR-770-5p potentiated the effectiveness of trastuzumab. Thus, regulating the expression level of miR-770-5p in combination with trastuzumab treatment may simultaneously inhibit the downstream elements of PI3K and MAPK signalling, thereby blocking the proliferation, motility and invasion capacities of HER2+ breast cancer cells.

## Introduction

Breast cancer is the most common malignancy in women, constituting approximately 30% of all cancer types [1]. Breast cancer is a heterogeneous disease with complex clinical behavior and responses to therapeutic intervention [2,3]. It is classified based on gene expression profiling, including HER2 positive (HER2+), luminal A or B, basal-like and presence of hormone receptors [4]. Approximately 70% of human breast cancers are estrogen receptor alpha positive (ER+), so anti-estrogen therapy is an effective treatment [5]. Tamoxifen citrate (TAM), which competes with the estrogen that binds to the estrogen receptor (ER), was the first selective estrogen receptor modulator (SERM) to be developed [6]. Tamoxifen has been used clinically for over 30 years as a partial agonist of ER to reduce the risk of recurrence and contralateral neoplasia in breast cancer treatment. However, the development of resistance to this drug is inevitable because of molecular crosstalk mechanisms in the tumor cells [7,8]. Additionally, HER2+ tumors, which constitute 25% of breast cancers, are also known to show resistance to tamoxifen and standard chemotherapeutic approaches [8–10]. Trastuzumab (Herceptin) is a FDA-approved recombinant humanized monoclonal antibody developed against the extracellular domain of the HER2 protein, which is currently used as a therapy for HER2-overexpressing breast cancer patients [11–14]. Elucidation of the molecular mechanism of trastuzumab treatment is therefore important as it may contribute to determining the resistance mechanisms of tumor cells to this drug.

MicroRNAs (miRNA), which are 20–25 nucleotides long, non-coding RNAs, are endogenous RNA molecules that are evolutionarily conserved and repress gene expression post-transcriptionally. These regulatory molecules play important roles in various cellular processes, such as differentation, cell growth and apoptosis. Since these processes are generally dysregulated in cancer, the relationship between miRNAs and cancer is quite important and solid [15]. miRNAs are deregulated in breast cancer and various types of other human cancers [11,15]. Since miRNAs may play effective roles in disease progression, they represent potential therapeutic targets for cancer as well. Modulating miRNA expression levels could provide effective diseases therapies [16,17].

miRNAs play regulatory roles in breast cancer progression and have the potential to reverse resistance to drugs like tamoxifen [18–20]. A few studies have investigated the relationship between drugs and miRNAs. One recently showed that miR-210 levels in plasma might be associated with trastuzumab resistance in patients [13]. Others found an effect of trastuzumab on the expression of miRNAs. However, these studies only focused on the oncogenic and tumor suppresor functions of individual miRNAs in trastuzumab sensitive or resistant cell lines [14–19] failing to explain the complexity of miRNA-mediated drug mechanisms.

In this study, we determined the expression profiles of miRNAs in tamoxifen and trastuzumab-sensitive breast cancer cell lines by qRT-PCR-array analysis to explain the common molecular mechanisms of these two drugs. Among the differentially expressed miRNAs, only one common miRNA, miR-770-5p, was responsive in a drug and cell line independent manner. Bioinformatics analysis, together with the experimental results, indicated that HER2 signaling was one of the targets of miR-770-5p. We showed that overexpression of miR-770-5p potentiated the effect of trastuzumab, especially in BT-474 cells. When miR-770-5p was overexpreesed in the presence of trastuzumab, there was downregulation in the total or phoshorylated levels of AKT and ERK. This downregulation of the major regulator proteins of PI3K and MAPK signaling may explain the potentiation mechanism of miR-770-5p in HER2+ cells.

## Compliance with ethical standards

Ethical approval: This article does not contain any studies with human participants or animals performed by any of the authors.

## Materials and methods

### Cell lines and culture

Three human breast cancer cell lines (BT-474, SK-BR-3, MCF-7) were obtained from the American Type Culture Collection and maintained in DMEM or McCoy medium supplemented with 10% FCS. All cell lines were cultured in humidified air supplemented with 5% CO_2_ at 37°C. The molecular characteristics of these cell lines are summarized in Table 1.

**Table 1:** Molecular classification of human breast cancer cell lines.

Cell lines Source	Subtype	Immunoprofile
BT-474	Luminal B	ER^+^, PR^+^, HER2^+^
SK-BR-3	HER2	ER^-^, PR^-^, HER2^+^
MCF-7	Luminal A	ER^+^, PR^+^, HER2^-^

### Tamoxifen or trastuzumab treatment and cell proliferation analysis

Tamoxifen (Cat. No: 54965-24-1), purchased from Tocris (Minneapolis, MN, USA), was dissolved in ethanol as a 100 μmol/ml stock solution. Trastuzumab (Herceptin) was obtained from Roche (Basel, Switzerland).

For the drug sensitivity test, two experimental designs were applied. First, MCF-7 and BT-474 cells were seeded at densities of 3×10^3^ and 6×10^3^ cells/ml respectively and cultured in 8 different concentrations of tamoxifen (100, 80, 40, 20, 10, 5, 2, 1 μmol/ml) for 3 days. Control cells were treated with the same concentrations of ethanol in the culture medium. For trastuzumab treatment, SK-BR-3 and BT-474 cells were plated in 96-well microtiter plates at a concentration of 6×10^3^/well, and cultured at 37°C in a humidified atmosphere with 5% CO_2_ overnight before treatments. The cells were incubated with decreasing trastuzumab concentrations (300, 60, 30, 6, 2, 0.5 and 0.1 μg/ml) for 3 days. Control groups received PBS at a concentration equal to that in the drug-treated cells.

Cell proliferation was measured using WST1 assay. Briefly, the cells were plated in 96-well plates at 8× 10^3^ per well in a final volume of 100 μl, before exposure to trastuzumab or tamoxifen at the aforementioned concentrations for 3 days. 10 ul of WST-1 reagent was added to each well on day 3. Incubation of the cells at 37°C for 3 h was followed by measuring absorbance at 480 nm on a Wallac Victor Counter (Perkin Ekmer, USA). The IC_50_ value, which represents the drug concentration required for 50% growth inhibition, was calculated with Graphpad Prism version 6.04 software (California, CA, USA).

### miRNA expression analysis by quantitative real-time PCR array

For miRNA profiling, a SYBR green-based miScript miRNA PCR Array (MIHS-3216ZG) was used (Qiagen). Endogenous controls, normalization controls, miRNA reverse transcription and positive controls were also tested for each array. After treatment of the cells with the effective concentrations of the drugs, total RNA was isolated using QIAzol reagent (Qiagen) according to manufacturer’s instructions, reverse transcribed to cDNA and used to measure miRNA expression. The plates were run on a Roche Light Cycler 480 instrument to analyze the expression of miRNAs using the obtained Ct values. The specificity of the miRNA assays was confirmed from the melting curves of the PCR products.

### Validation of real-time PCR array

Mature miR-770-5p levels were quantified with Qiagen miRNA assays. Quantitative PCR was performed using SYBR mix (Qiagen) on a Roche Light Cycler 480. U6 snRNA (MS00033740) was used as the internal control (Qiagen). Relative expression levels were calculated using the 2^-ΔCT^ method while fold changes were calculated using the equation 2^-ΔΔCT^.

### Transfection with miRNA modulator

4x10^5^ cells were seeded in six-well plates and transfected with the effective concentration, 25 nM, of either the miRNA mimic (MSY0003948) or the negative scrambled control (scr, SI03650318) using HiPerFect Transfection Reagent (301705) (Qiagen, Germany). After an incubation period of 48 h or 72 h, cells were harvested using a cell scraper in ice-cold PBS. The RNA and protein were isolated for further qPCR and western blot analysis respectively.

Viability of breast cancer cells after transfection of miR-770-5p miRNA mimic was measured by the same WST1 protocol.

The synergistic effect of trastuzumab with miR-770-5p was also analyzed. For this purpose, cells were treated with miR-770-5p mimic or scrambled control RNA with varying doses of the drug for 48 h. After incubation, the same WST1 protocol was applied to the cells.

### Wound healing assay

For the wound healing assay, cells (5 × 10^5^/well) were seeded into six-well plates and cultured under standard conditions. When the cells reached confluency, they were transfected with miR-770-5p mimic or the scrambled control. A wound was performed with a 20 uL tip on confluent BT-474, SK-BR-3 and MCF-7 cells (t_0_). We observed and photographed the cells with a microscope at 0, 24, 48 and 72 hours after transfection. Gap widths at t_0_ and t_final_ were measured and the average gap width ratio of miR-770-5p transfected cells was normalized to that of control cells using Tscracth [21].

### Invasion assay

Cell invasion analysis was performed with xCELLigence real-time cell analyser. At 24h post-transfection with miRNA mimic, 4x10^4^ BT-474 and SK-BR-3 cells in 200 uL of serum-free medium were seeded into the upper chamber of the ACEA Biosciences Inc. CIM-plate wells (Cat. No: 2801038), fitted with a microporous membrane of matrigel separating the upper and lower chamber. The lower chamber was filled with culture medium supplemented with 10% FBS as a chemoattractant. Cell invasion was monitored for 24 hours with xCELLigence real-time cell analyser, using CIM-plate and measuring impedance-based signals.

### Western blot analysis

Cells in culture were lysed using Complete Lysis-M kit (Roche). The protein concentrations of the lysates were quantified using Coomassie Plus Protein Assay Reagent (Thermo Scientific). 10 ug of protein for each sample were loaded on to 8% SDS-PAGE gel. Separated proteins were transferred to a PVDF membrane (L-08008-001, Advansta) in wet transfer buffer. Membranes were blocked with 5% milk in TBST (0.5%) for 1 hour at room temperature before incubation at +4°C overnight with the following antibodies: HER2 (1:1000, ab8054, Abcam), total AKT (1:1000, sc8312, SantaCruz), phospho-AKT (Ser 473, 1:1000, sc-7985-R, SantaCruz), total ERK2 (1:1000, sc-154, SantaCruz), phosho-ERK 1/2 (Thr 202 / Tyr 204, 1:1000, sc-81492, SantaCruz) and beta-actin (1:1000, 634801, Biolegend) in 3% milk powder-TBST. After incubation with HRP-conjugated secondary antibodies, the protein bands were detected using WesternBright Sirius Kit (K-12043-D20, Advansta).

### Target prediction and pathway analysis

Predicted miRNA targets were retrieved from the mirWalk2.0 target prediction tool, which collects data from 12 different programs (mirWalk, miRDB, PITA, MicroT4, miRMap, RNA22, miRanda, miRNAMap, RNAhybrid, miRBridge, PICTAR2, TargetScan) [22]. To assess the functional enrichment of the gene list, we used Webgestalt (WEB-based GEne SeT AnaLysis Toolkit) [23].

### Statistical analysis

All the experiments were performed with a minimum of two biological and two technical replicates each. Student’s t-test was performed to test the differences, which were considered to be statistically significant at a p-value of less than 0.05 between two samples. The nonlinear regression (curve fit) method was used to analyze dose-response data for mimic transfection with drug only or drug plus miR-770-5p.

## Results

### Identification of tamoxifen or trastuzumab-responsive microRNAs

The putative roles of miRNAs in tamoxifen or trastuzumab responses were investigated by miRNA qRT arrays to search for differentially-expressed miRNAs between three different breast cancer cell lines.

**Fig 1:**
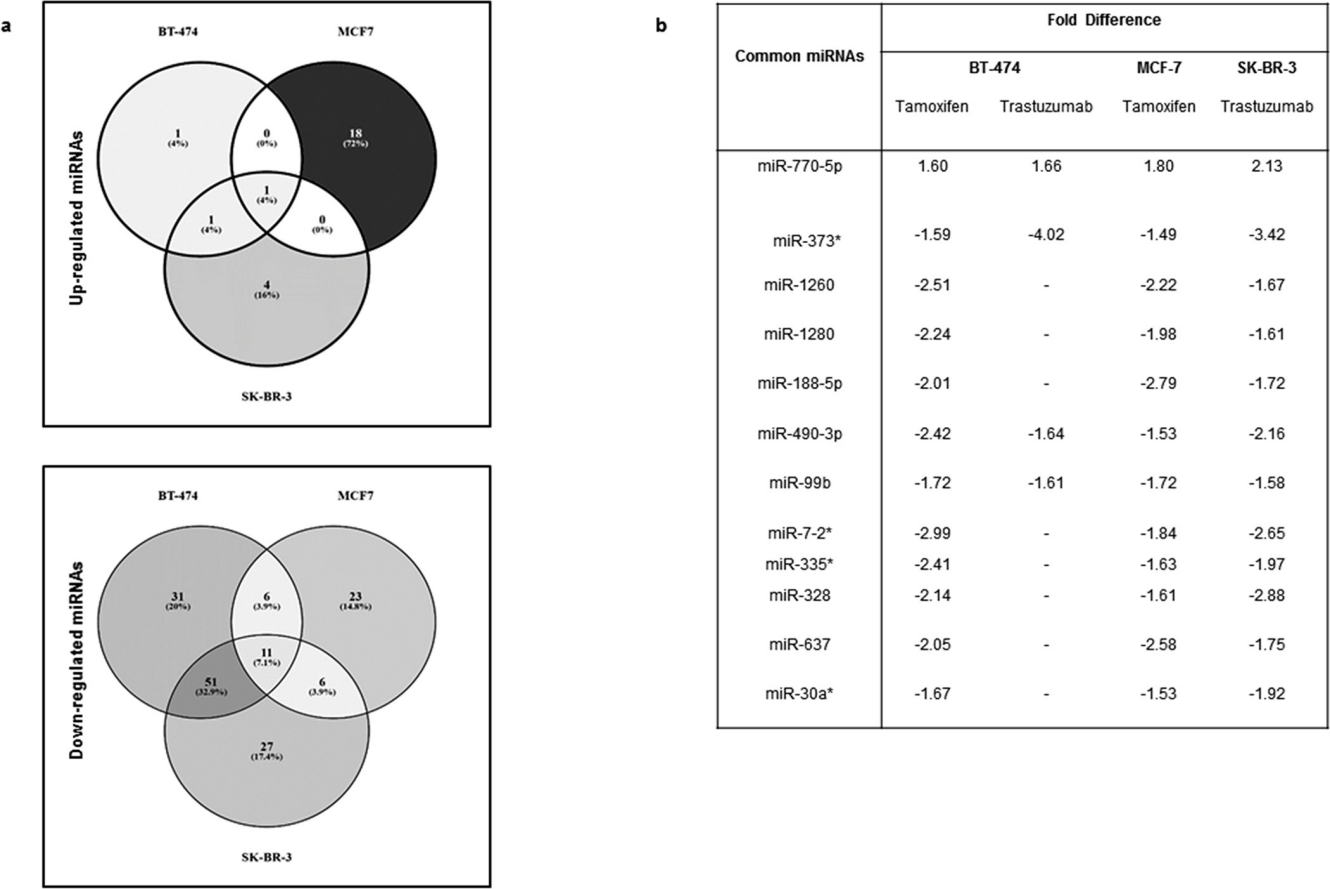
Trastuzumab and Tamoxifen responsive miRNAs were identified by qRT-PCR Array. **(a)** HER2+ and ER+ BT-474 cells were treated with both tamoxifen and trastuzumab while ER+ MCF-7 cells and HER2+ SK-BR-3 cells were only tretated with tamoxifen and trastuzumab respectively. There was only one common responsive miRNA among upregulated miRNAs; 11 miRNAs were commonly downregulated in all cell lines. The common responsive miRNAs are listed in b.

The miRNA qRT array results showed that, for trastuzumab treatment, 53 miRNAs were differentially expressed in BT-474 cells (3 upregulated, 50 downregulated) while there were 101 DE miRNAs for SK-BR-3 cells (6 upregulated, 95 downregulated) (S1 Table). Those miRNAs that showed more than 1.5-fold difference in expression level at p<0.05 were selected as DE miRNAs. When the DE miRNA lists for the two cell lines were intersected, 64 miRNAs were found to be commonly responsive to trastuzumab, of which 62 downregulated and 2 upregulated (Fig 1A). The array analysis results indicated that 65 and 68 miRNAs were found to be tamoxifen-responsive in MCF-7 and BT-474 cells respectively (S1 Table). Among the responsive miRNAs, 19 were upregulated in MCF-7 cells while 47 were downregulated. For BT-474 cells, out of 68 DE miRNAs, 2 were upregulated and 66 were downregulated. DE miRNAs in tamoxifen-treated MCF-7 and BT-474 cells were compared with VENNY and 17 common downregulated miRNAs and 1 common upregulated miRNAs were detected (Fig 1A). When all the DE expressed lists were intersected, miR-770-5p was significantly responsive in a cell and drug-independent manner with consistent upregulation (Fig 1B).

### Targets of miR-770-5p participate in cancer-related pathways

Having determined that miR-770-5p was a common drug target in each cell line, we wanted to further characterize its potential molecular function. Through the use of the target prediction software mirWalk2.0 [20], we identified the predicted targets of miR-770-5p. Pathway enrichment anlysis results showed that the target genes enriched significantly in pathways related to cancer progression, such as ErbB, Insulin and MAPK signaling pathways (Table 2).

**Table 2:** Pathway enrichment analysis results conducted with the targets of miR-770-5p.

KEGG Pathway Name	Number of Genes	Statistics
ErbB Signaling Pathway	47	P = 3.67e-18
Calcium signaling pathway	71	P = 8.01e-18
Insulin signaling pathway	64	P = 6.69e-20
Focal adhesion	91	P = 2.84e-27
MAPK signaling pathway	109	P = 2.27e-27

**Fig 2:**
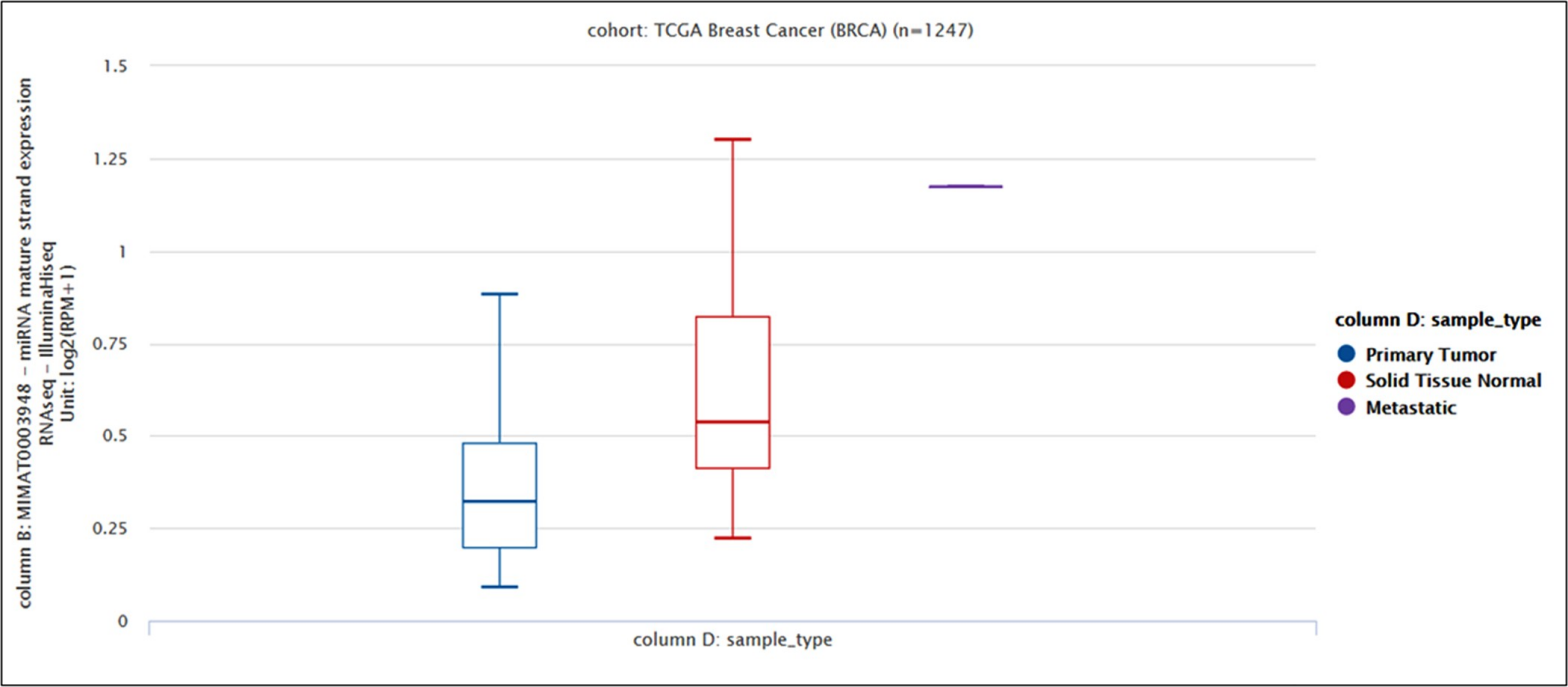
Lower expression of miR-770-5p is associated with tumor samples. Expression level of miR-770-5p was downregulated in tumor samples compared to normal samples (One-way ANOVA; p<0.00005621, f = 10.44).

Additionally, we assessed miR-770-5p expression level in breast tumor tissues in TCGA breast cancer BRCA (n = 1247) cohort using XenaBrowser [24]. miR-770-5p expression was significantly downregulated in tumor samples compared to normal samples (f = 10.44, p< 0.00005621) (Fig 2), which led us to upregulate the expression of miR-770-5p in tumor cells as a strategy to explore its molecular function in cancer cells.

### Motility and invasion are regulated by miR-770-5p

**Fig 3:**
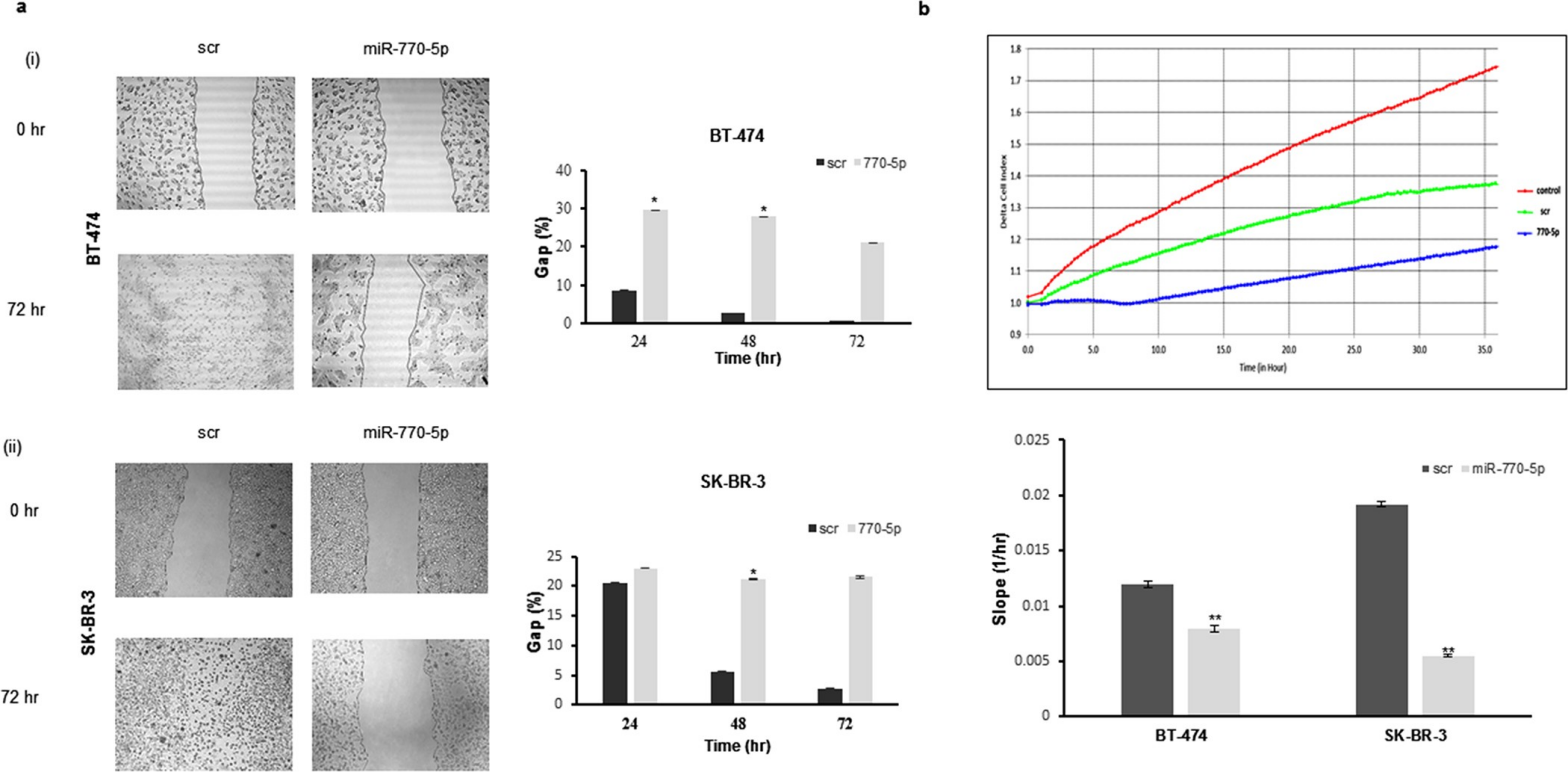
miR-770-5p regulates motility and invasion in BT-474 and SK-BR-3 cells. **(a)** The rate of motility was assessed by wound-healing assay. Wound closure was observed in scrambled control-transfected cells in a time-dependent manner while miR-770-5p mimic transfected cells lost their motility (n = 2, *p<0.005). **(b)** Cell invasion analysis was performed by xCELLigence real-time cell analyzer measuring impedance-based signals. Cell invasion capacity decreased in both of the cells transfected with miR-770-5p mimic compared to scrambled control-transfected cells (n = 2, **p<0.0001).

To determine the effect of miR-770-5p overexpression on cell motility, wound-healing assays were performed after mimic transfection. The wound closure in the scratched was quantitated over 72 hours. miR-770-5p overexpression decreased the cells’ motility capacity compared to scrambled control-transfected cells. Wound closure was 100% in both BT-474 and SK-BR-3 cells when transfected with negative control. However, it reduced to 20% in both cell lines in a miR-770-5p overexpression-dependent manner (Fig 3A). Additionally, monitoring with xCELLigence-based invasion assay revealed that miR-770-5p overexpression decreased the cells’ invasion kinetics (Fig 3B). Assessment of the proliferative activity of the mimic-transfected cells showed that miR-770-5p did not affect cell proliferation by itself (S1 Fig).

### Upregulation of miR-770-5p changes cellular response to trastuzumab in HER2 (+) breast cancer cells

**Fig 4:**
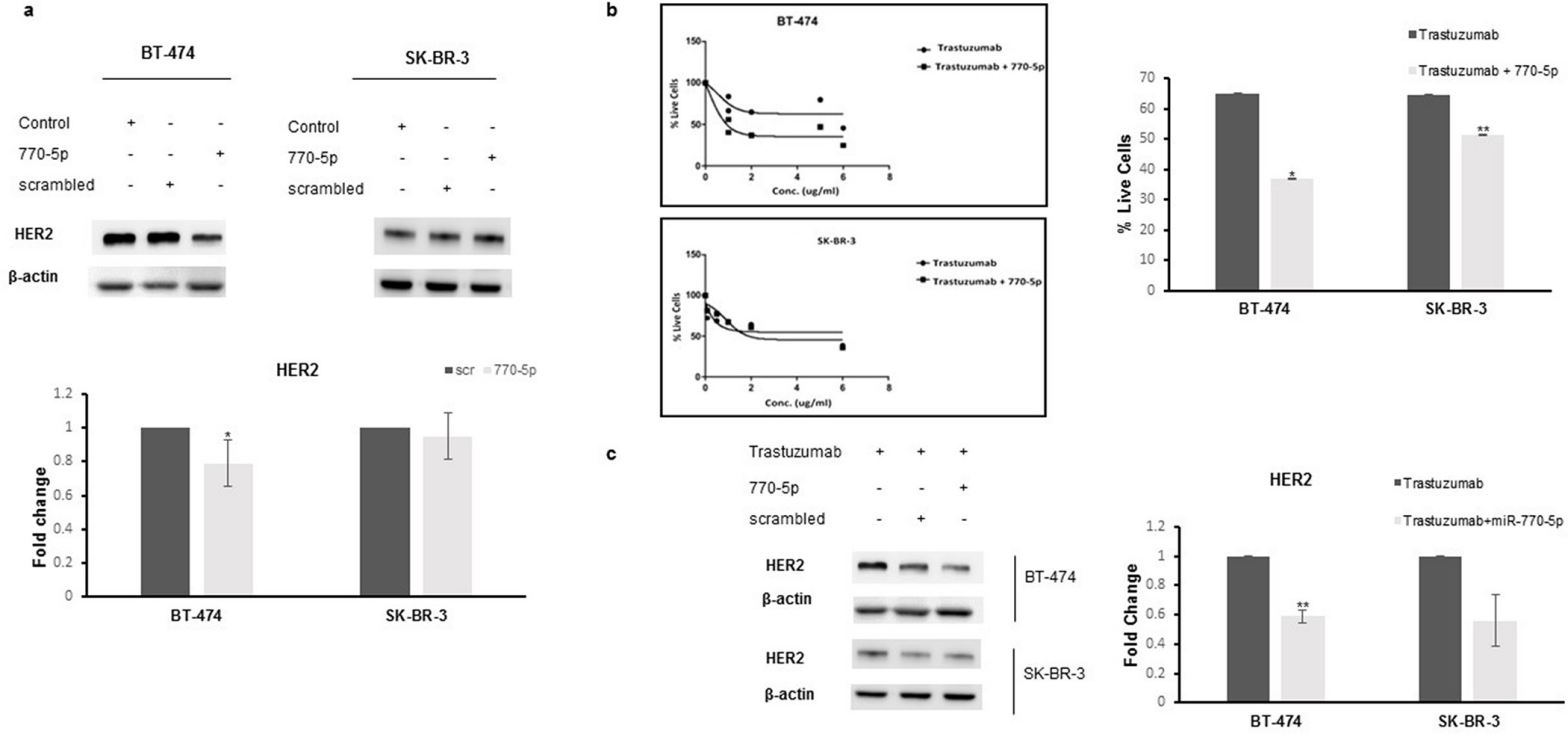
miR-770-5p overexpression downregulated HER2 and increased the effect of trastuzumab. **(a)** The protein expression level of HER2 was slightly diminished in both cell lines following miR-770-5p transfection n = 3, p<0.05). **(b)** Combinational treatment of cells with trastuzumab and miR-770-5p decreased cell viability in BT-474 and SK-BR-3 cells (n = 3, *p<0.001, **p<0.05); HER2 expression also reduced significantly, especially in BT-474 cells **(c)** (**p<0.05).

The pathway enrichment analysis results suggested that miR-770-5p might function as a mediator of the HER2 signaling pathway (Table 2). Furthermore, according to the mirWalk results, HER2 was predicted to be regulated by miR-770-5p. Supporting this prediction, we demonstrated that upregulation of miR-770-5p slightly decreased the expression level of HER2 protein in BT-474 cells (Fig 4A). To clarify whether miRNA upregulation in cells by mimic transfection increases the growth-inhibitory effect of trastuzumab, the sensitivity of HER2 overexpressing cells (BT474 and SKBR3) to trastuzumab treatment was analyzed by WST1 assay after miR-770-5p mimic transfection. This showed that the effect of trastuzumab increased with upregulation of miRNA in the cells by decreasing the percentage of living cells from 60% to 40% in BT-474 cells and from 60% to 50% in SK-BR-3 cells (Fig 4B). In addition, HER2 protein levels decreased significantly in both cell lines when the cells were treated with a combination of trastuzumab and miR-770-5p. That is, miR-770-5p potentiated the effect of trastuzumab in HER2+ cells (Fig 4C).

### miR-770-5p regulates cancer cell behavior by targeting several genes involved in the HER2 signaling pathway

From the bioinformatics analysis, HER2 was predicted to be the target of miR-770-5p and our protein results supported the regulative effect of miR-770-5p on HER2. To explore the role of miR-770-5p on the HER2 signaling pathway, downstream elements were assesed in miR-770-5p-overexpressing BT-474 and SK-BR-3 cells. The total or phoshorylation states of AKT and ERK, which are major regulator proteins of PI3K and MAPK signaling respectively, were examined.

**Fig 5:**
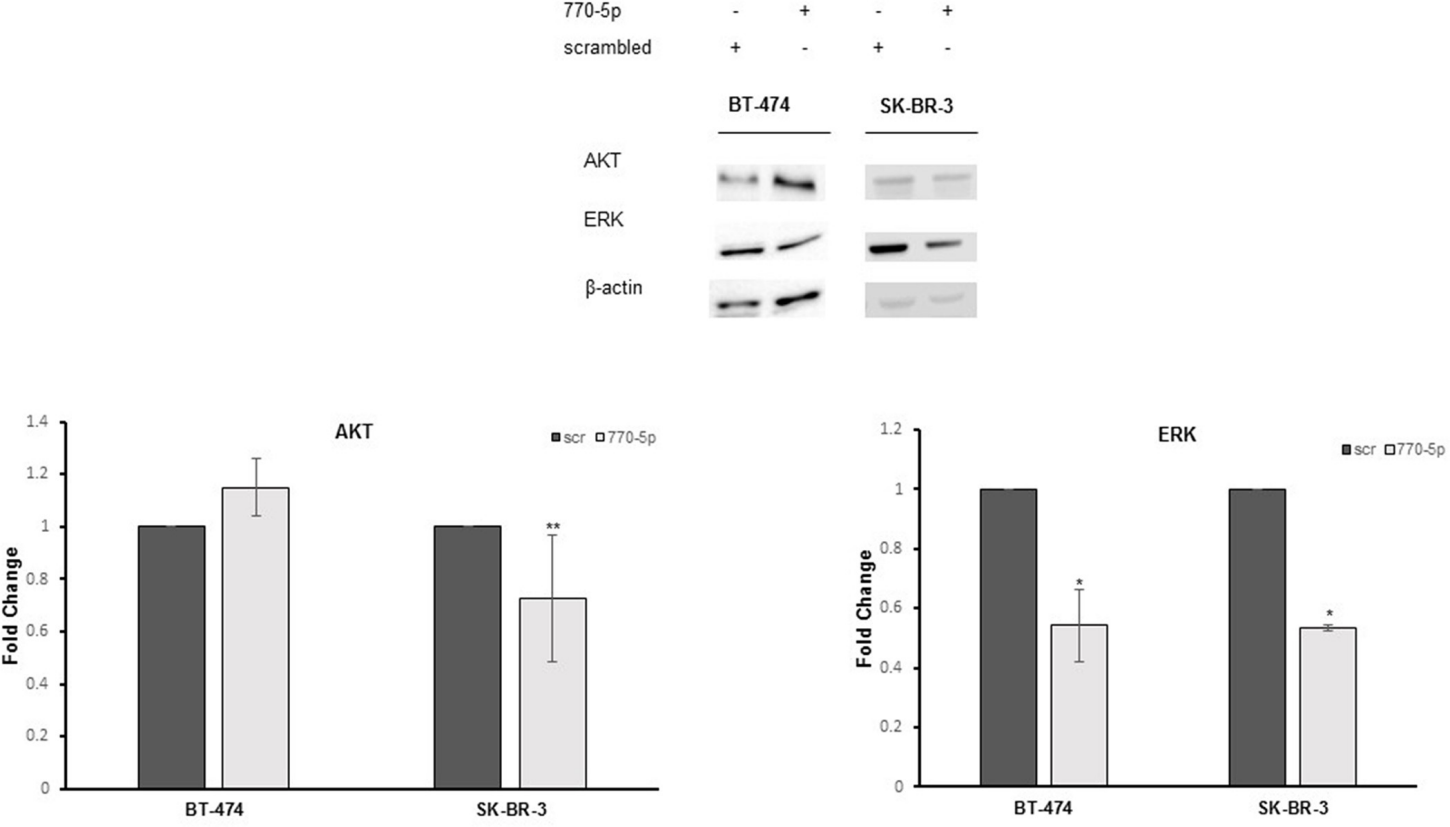
miR-770-5p targeted total ERK in BT-474 and SK-BR-3 cells. Protein expression of total AKT and ERK were detected in BT-474 and SK-BR-3 cells transfected with miR-770-5p mimics and scrambled control (scr). ERK expression decreased in BT-474 cells after mimic transfection (n = 4, *p<0.01). Both the ERK expression and the AKT expression, decreased significantly only in SK-BR-3 cells (n = 2, **p<0.05).

**Fig 6:**
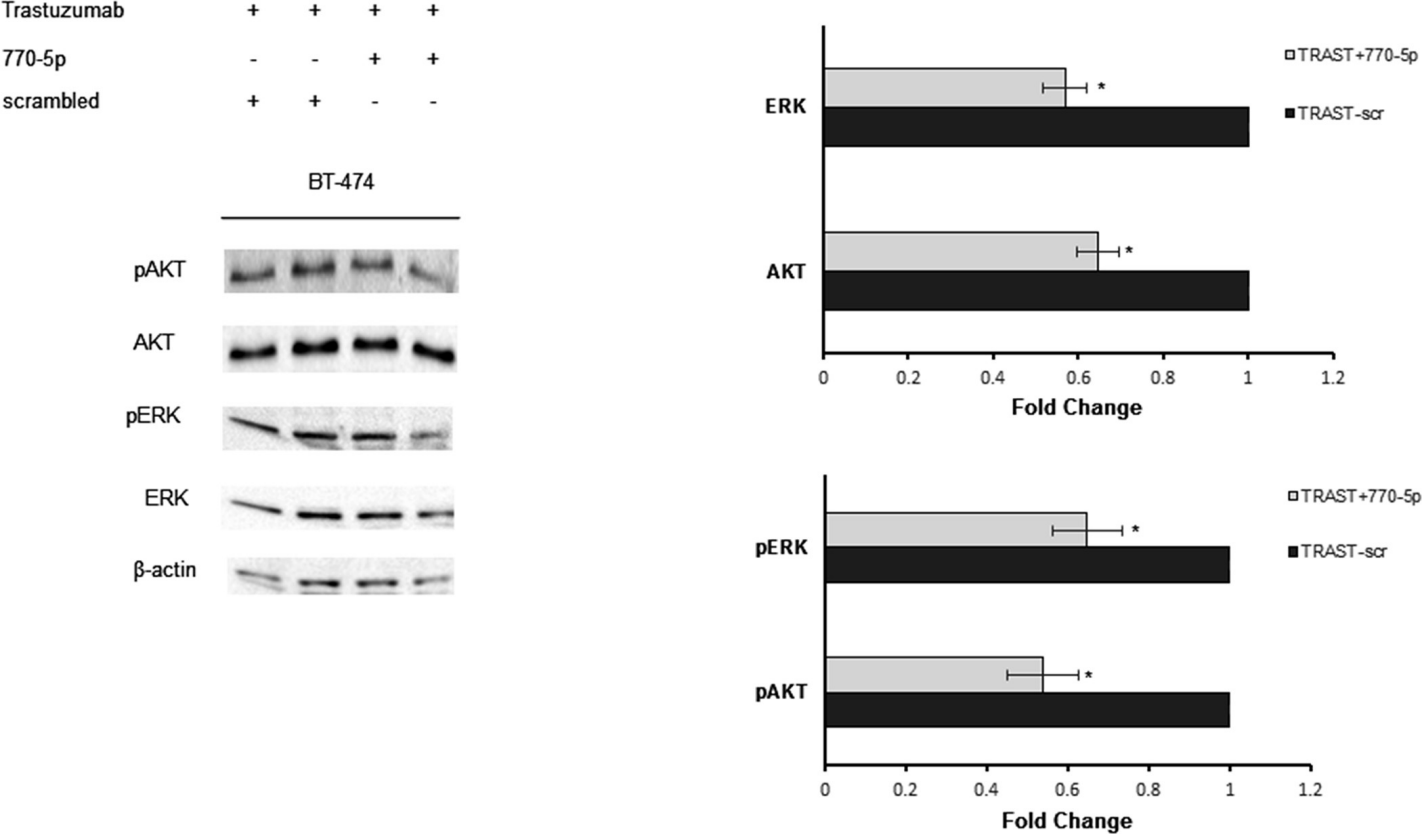
Trastuzumab treatment together with miR-770-5p modulates AKT and ERK expression level. miR-770-5p mimic was transfected to BT-474 cells and; total and phoshorylated AKT and ERK protein levels were analyzed by Western blot 72 h after transfection. Combination of trastuzumab with miR-770-5p mostly affected pERK and pAKT levels in BT-474 cells (n = 3, *p<0.05).

The protein analysis results showed that total ERK expression level was downregulated in both cell lines through miR-770-5p overexpression alone compared to control cells, while total AKT level was only diminished significantly in SK-BR-3 cells (Fig 5; t-test, p<0.05). Hence, the expression levels of total and phosphorylated forms of AKT and ERK proteins were analysed in BT-474 cells to determine the response to trastuzumab combined with miR-770-5p. In the presence of trastuzumab, total protein levels of both AKT and ERK were downregulated while p-AKT and p-ERK levels also decreased significantly (Fig 6; t-test, p<0.05).

## Discussion

In this study, we identified a common miRNA response in cells expressing ER and HER2 to two different drugs, tamoxifen and trastuzumab. According to the qRT-PCR-array results, miR-770-5p was significantly responsive, independent of cell and drug type, with consistent overexpression. miR-770-5p has been linked to various cancers, such as gastric cardia adenocarcinoma [25], ovarian cancer [26], non-small cell lung cancer [27] and hepatocellular carcinoma [28]. It has also been shown to be downregulated in breast cancer. A recent study reported that miR-770-5p is downregulated in chemo-resistant triple negative breast cancer (TNBC) tissues while its ectopic expression antagonized resistance and metastases by targeting *STMN1* [29]. One of the deregulated miRNAs may be a potential biomarker to predict clinical outcomes in TNBCs recieving neoadjuvant chemotherapy [30]. However, the role of miR-770-5p in ER+ and HER2+ breast cancer cells, and its response to tamoxifen and trastuzumab treatment has not previously been determined. Furthermore, our TCGA data analysis, which indicates its significant downregulation in breast tumors compared to normal breast tissues, supports its possible clinical importance and led us to conduct further functional analysis.

In the pathway enrichment analysis, targets of miR-770-5p were significantly enriched in focal adhesion, MAPK and ErbB signaling pathways, which are closely related with motility and invasion [31]. In parellel with this bioinformatics analysis, the motility and invasion capacity of both HER2+ cells decreased with the overexpression of miR-770-5p. This decrease in motility and invasion may be down to ERK downregulation, which is one of the putative targets of miR-770-5p [32,33].

Since HER2 has been identified as one of the targets of miR-770-5p, and pathway enrichment analysis conducted with putative targets of miR-770-5p indicated that ErbB signaling is one of the regulated pathways, we focused on this and a downregulation of HER2 protein was observed, especially in miR-770-5p-restored BT-474 cells. To follow the downstream signal through PI3K and MAPK signaling [34], we examined the expression levels of two major regulator proteins, AKT and ERK. Although total ERK expression was downregulated in both cell lines, total AKT downregulation was only observed in SK-BR-3 cells. This can be explained by variable mutation profiles as well as the different receptor status of two cell lines. c.333G>C (p.K111N) mutation in *PIK3CA* gene in BT-474 cells is well defined, which could explain the stable expression level of AKT in miR-770-5p transfected cells [35]. Finally, this stability could be due to the crosstalk mechanism between HER2 and growth factor receptors [10,36]. It was previously shown that complete response rates to trastuzumab-based treatment are lower in patients with the highest ER expression levels in HER2-positive breast cancers, like ER and HER-positive BT-474 cells, compared to ER-negative and HER2-positive SK-BR-3 cells [37]. In support of this, when cells were transfected with miR-770-5p mimic in the presence of trastuzumab, we observed downregulation in total AKT and ERK expression as well as their phosphorylated forms compared to scrambled control-transfected cells in both cell lines. Multiple levels of crosstalk are present between the PI3K/Akt and MAPK signaling pathways, which can compensate for each other [38,39]. Hence, miR-770-5p may block this crosstalk in the presence of trastuzumab, thereby strengthening the antiproliferative effect of trastuzumab.

Our qRT-PCR-array data show that miRNA expression levels in tumor cells can be potent in therapeutic response, and several preclinical studies have assessed the combination of miRNA-based therapeutics with chemotherapy [40]. Baldasarri et al. demonstrated that a combination treatment with miRNAs *in vitro*, especially miR-126, miR-9, miR-181a and miR-326, magnify the activity of specific breast cancer drugs [41]. In another study, restoring miR-375 sensitized cells to tamoxifen. This indicates it to be a potential target for treatment-resistant breast cancer [19]. miR-218 targeted *BRCA1* to sensitize breast cancer cells against cisplatin [42] and miR-542-3p silencing restored trastuzumab resistance via PI3K-AKT pathway regulation in breast cancer cells [43]. These data obtained from preclinical studies provide insights for using miRNA-based therapies to improve the effectiveness and potentiate the anticancer activity of drugs by regulating proliferation, motility or invasion [44]. One strategy to improve treatment effectiveness in HER2-overexpressing cancers, particularly those that develop resistance to HER2-targeted therapies, could be combined inhibition of PI3K and MEK. In conclusion, we showed that miR-770-5p downregulated AKT and ERK through HER2 signaling and potentiated the activity of trastuzumab in BT-474 cells. Here, we could suggest that mediating miR-770-5p in combination with trastuzumab treatment could inhibit two important pathways simultaneously, thereby blocking the proliferation, motility and invasion capacities of HER2+ breast cancer cells (S2 Fig).

A combination of miR-770-5p with thyrosine kinase inhibitors could also make breast cancer treatments more effective. However, preclinical animal models are needed to confirm the effectiveness of these combined therapies *in vivo*.

## Supporting information

S1 Table Differentially expressed miRNA lists. (XLSX)

S1 Fig Viability of the cells after miR-770-5p transfection. Although the viability of the cells decreased significantly in miR-770-5p mimic-transfected BT-474 and SK-BR-3 cells compared to scrambled control-transfected cells, the total viability of the cells diminished only 20% and 10% for BT-474 and SK-BR-3 cells respectively (n = 2, *p<0.02).
(TIF)

S2 Fig Proposed action mechanism of miR-770-5p in HER2 signaling. miR-770-5p is shown to regulate HER2 signaling by targeting HER2, AKT and ERK. Introducing miR-770-5p may reduce the expression of HER2 and in the presence of trastuzumab it may downregulate AKT and ERK that potentiate the activity of trastuzumab.
(TIF)

S3 Fig Western blot images. Original uncropped blots of Figs 4, 5 and 6.
(PDF)

## Abbreviations

BC: Breast Cancer
DE: Differentially Expressed
ER: Estrogen Receptor
ER+: Estrogen receptor alpha positive
HER2+: HER2 positive
HER2: Human Epidermal Growth Factor Receptor 2
MBC: Metastatic breast cancer
miRNAs: MicroRNAs
PR: Progesterone Receptor
TAM: Tamoxifen citrate
TGCA: The Cancer Genome Atlas
TNBC: Triple Negative Breast Cancer
