# Supplementary figures and images for: Involvement of miR-770-5p in trastuzumab response in HER2 positive breast cancer cells
Source: PLoS One. 2019 Apr 22;14(4):e0215894. doi: 10.1371/journal.pone.0215894 (PMC6476517; doi:10.1371/journal.pone.0215894)

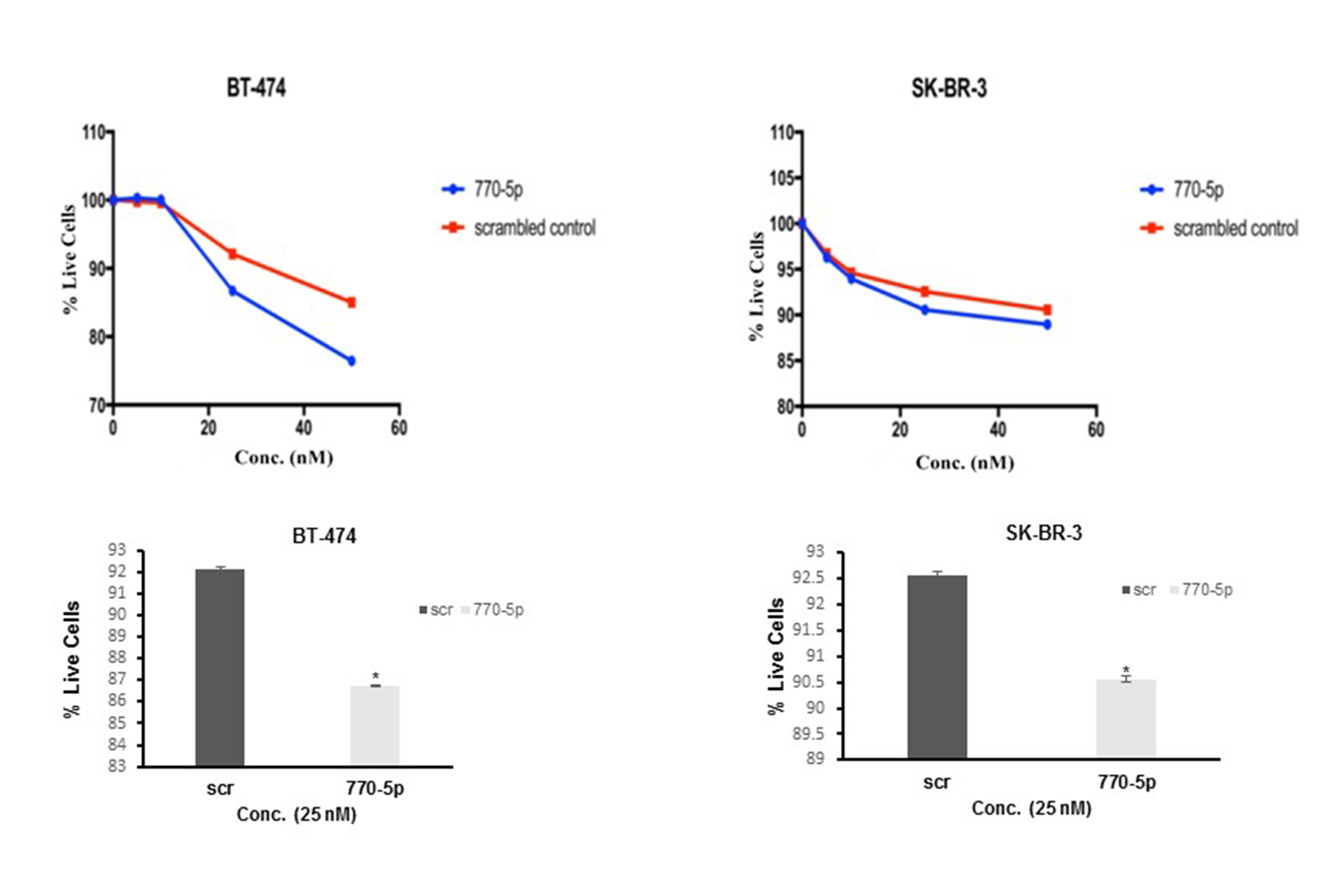

Supplement: S1 Fig — Although the viability of the cells decreased significantly in miR-770-5p mimic-transfected BT-474 and SK-BR-3 cells compared to scrambled control-transfected cells, the total viability of the cells diminished only 20% and 10% for BT-474 and SK-BR-3 cells respectively (n = 2, *p<0.02). (TIF) [file pone.0215894.s002.tif]

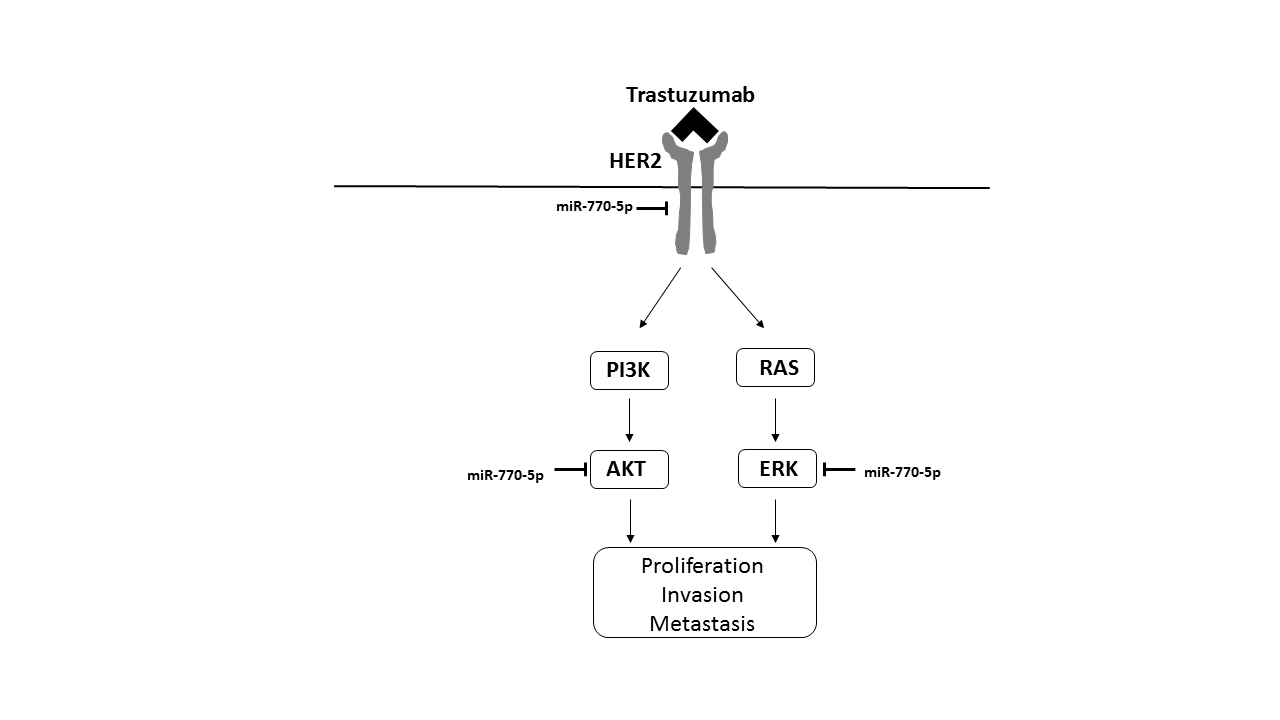

Supplement: S2 Fig — miR-770-5p is shown to regulate HER2 signaling by targeting HER2, AKT and ERK. Introducing miR-770-5p may reduce the expression of HER2 and in the presence of trastuzumab it may downregulate AKT and ERK that potentiate the activity of trastuzumab. (TIF) [file pone.0215894.s003.TIF]

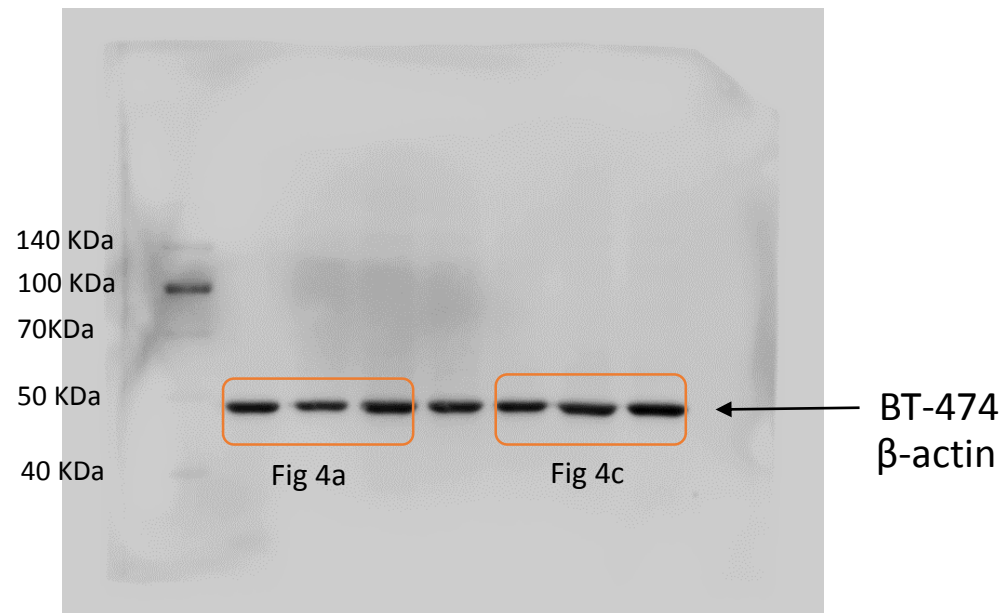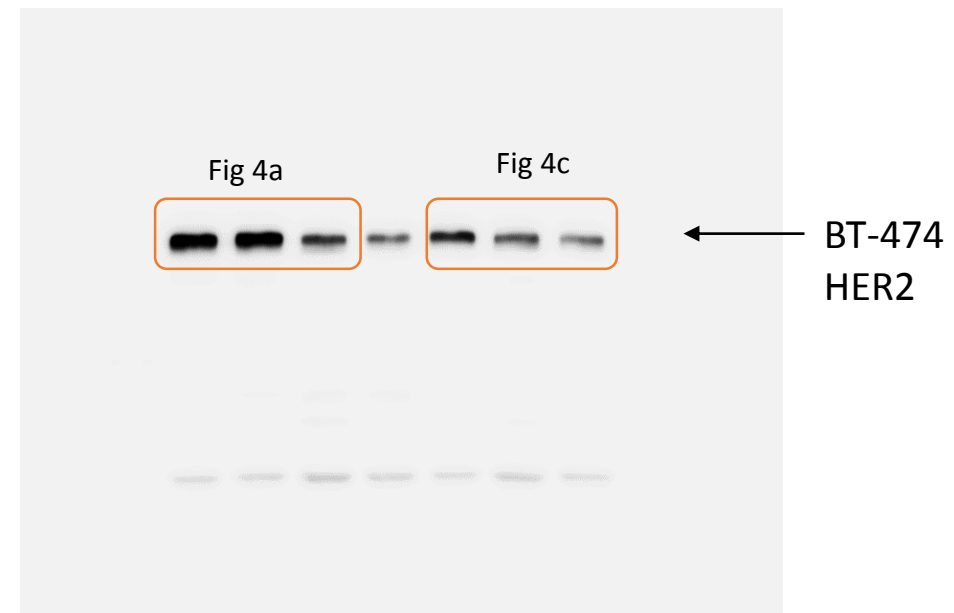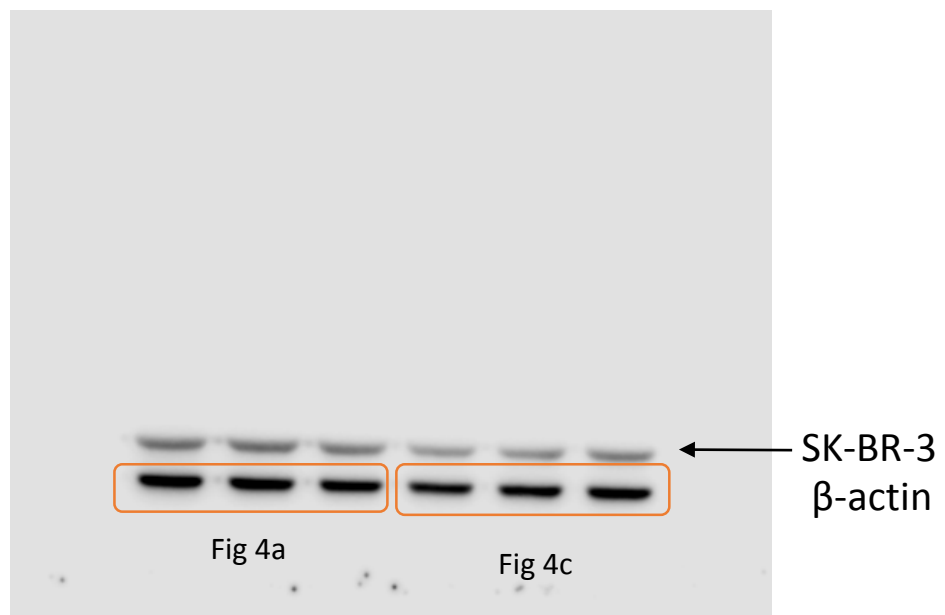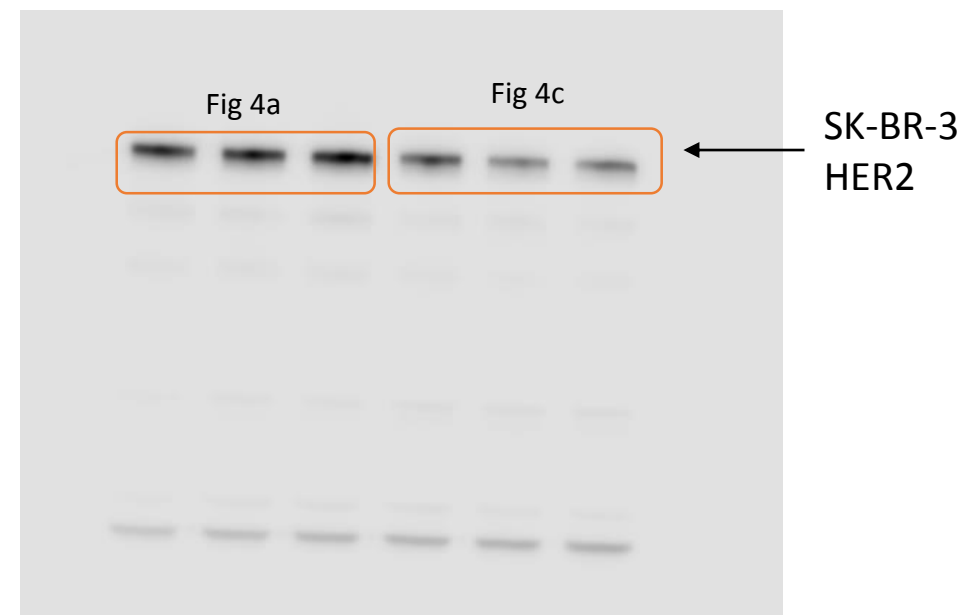

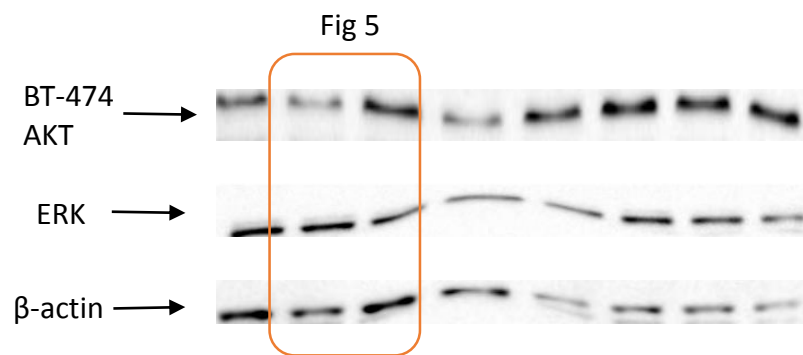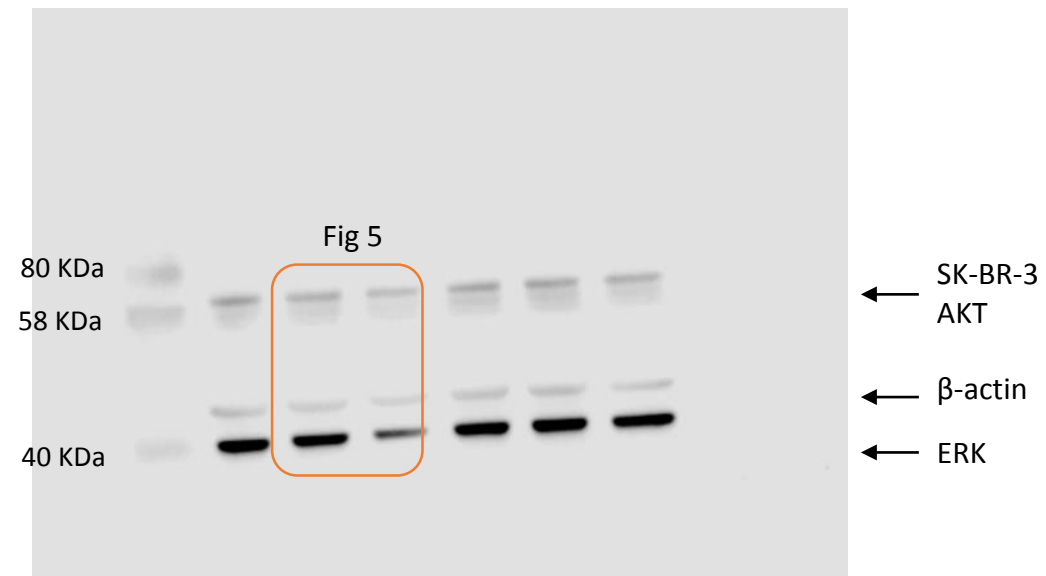

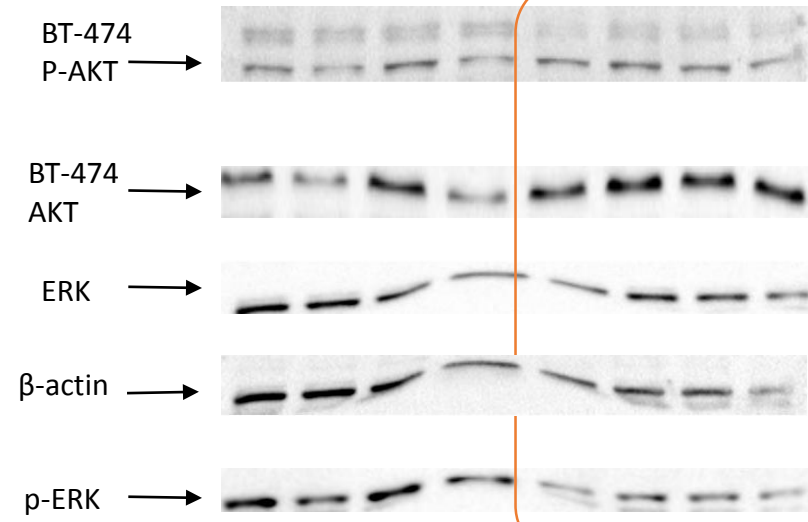

Supplement: S3 Fig — Original uncropped blots of Figs 4, 5 and 6. (PDF) [file pone.0215894.s004.pdf]
